# Neutralizing Antibody Fails to Impact the Course of Ebola Virus Infection in Monkeys

**DOI:** 10.1371/journal.ppat.0030009

**Published:** 2007-01-19

**Authors:** Wendelien B Oswald, Thomas W Geisbert, Kelly J Davis, Joan B Geisbert, Nancy J Sullivan, Peter B Jahrling, Paul W. H. I Parren, Dennis R Burton

**Affiliations:** 1 Departments of Immunology and Molecular Biology, The Scripps Research Institute, La Jolla, California, United States of America; 2 United States Army Medical Research Institute of Infectious Diseases, Fort Detrick, Maryland, United States of America; 3 Vaccine Research Center, National Institutes of Health, Bethesda, Maryland, United States of America; Washington University School of Medicine, United States of America

## Abstract

Prophylaxis with high doses of neutralizing antibody typically offers protection against challenge with viruses producing acute infections. In this study, we have investigated the ability of the neutralizing human monoclonal antibody, KZ52, to protect against Ebola virus in rhesus macaques. This antibody was previously shown to fully protect guinea pigs from infection. Four rhesus macaques were given 50 mg/kg of neutralizing human monoclonal antibody KZ52 intravenously 1 d before challenge with 1,000 plaque-forming units of Ebola virus, followed by a second dose of 50 mg/kg antibody 4 d after challenge. A control animal was exposed to virus in the absence of antibody treatment. Passive transfer of the neutralizing human monoclonal antibody not only failed to protect macaques against challenge with Ebola virus but also had a minimal effect on the explosive viral replication following infection. We show that the inability of antibody to impact infection was not due to neutralization escape. It appears that Ebola virus has a mechanism of infection propagation in vivo in macaques that is uniquely insensitive even to high concentrations of neutralizing antibody.

## Author Summary

Ebola virus is one of the most feared of human pathogens with a mortality that can approach 90% and an extremely rapid disease course that can lead to death within days of infection. Antibodies able to inhibit viral infection in culture, neutralizing antibodies, can typically prevent viral infection in animals and humans when present prior to infection, at sufficient concentration. Such neutralizing antibodies may be provided through passive administration or induced by vaccination. We have previously shown that a human neutralizing antibody can protect guinea pigs against Ebola virus. However, here we show that this antibody does not protect monkeys against Ebola virus and surprisingly appears to have very little impact upon the rapid course of infection, despite being present at very high levels in the blood of the monkeys. We conclude that administering antibody prior to or immediately following exposure to Ebola virus, for example, after an accident in a research setting or a bioterrorist attack, is unlikely to be effective in preventing disease. Recent successes in protecting monkeys against Ebola virus through vaccination may be independent of antibody, or, more likely, critically dependent on the cooperation of antibody and cellular immunity.

## Introduction

*Editor's note: The potential efficacy of pre- and post-exposure prophylaxis against Ebola virus infection, as well as the fundamentally important question of whether neutralizing bodies are important for Ebola virus resistance, is addressed by a related manuscript in this issue of* PLoS Pathogens. *Please see doi:10.1371/journal.ppat.0030002 by Feldmann et al.*

Passive transfer of relatively high concentrations of neutralizing antibodies can protect against challenge with a range of viruses in animal models and in humans [1–3]. Protection in some cases is in the form of sterilizing immunity, i.e., no viral replication is observed following challenge [2,4,5]. In other cases (e.g., [5,6]), some replication is observed but protection from disease is achieved, presumably because neutralizing antibody sufficiently blunts infection for T cell and innate immunity to resolve infection [7]. It might be expected that passive neutralizing antibody would be most effective against challenge with acute viruses. Many acute viral infections are resolved even in the absence of neutralizing antibody, and the blunting effect of passive antibody would provide more time for the development of effective cellular immune responses. In contrast, chronic viruses may present a greater challenge to passive antibody, since, in the absence of sterilizing immunity, there is a window of opportunity for the virus to establish a chronic infection before cellular immunity can be mobilized.

Ebola virus (EBOV) causes a severe acute infection in humans [8]. Infection with the Ebola Zaire strain, Zaire ebolavirus (ZEBOV), produces mortality in the range of 60%–90% [9] with death generally occurring around 7–11 d following the appearance of symptoms [8]. There is a single report describing the use of convalescent sera to treat EBOV infection [10]. However, the patients in this report may have already been through the worst stages of the disease, and it is not clear that serum antibodies were responsible for their recovery [10]. Further, neutralizing antibody titers in survivors of EBOV infection tend to be rather low, although we have isolated a neutralizing human monoclonal antibody (mAb), KZ52, of good potency from a convalescent individual [11].

The ability of passive antibody to protect against EBOV has been investigated in a number of animal models. The guinea pig and mouse models use EBOVs that have been serially passaged to adapt to replication in the respective animals and are highly lethal. Protection has been demonstrated in the guinea pig model using neutralizing horse, sheep, and goat immunoglobulin G (IgG) against EBOV [12,13] and the human anti-EBOV GP mAb, IgG KZ52. This antibody neutralizes ZEBOV (1995, Kikwit) with a 50% inhibitory concentration (IC_50_) of 0.05–0.3 μg/ml and an IC_90_ of 0.5–2.6 μg/ml in Vero cells [11,14] and an IC_50_ of approximately 0.05–1 μg/ml and a IC_90_ of 0.5–2 μg/ml in primary human monocytes/macrophages [14]. We showed that when administered subcutaneously at a dosage of 25 mg/kg up to 1 h after challenge, the antibody protects against robust ZEBOV challenge (10,000 plaque-forming units [pfu]) in the guinea pig model [6].

Macaques provide a model of EBOV infection that is likely closer to human infection. The human virus can be used directly in macaques without need for adaptation and the course of disease mirrors that seen in humans [8]. In cynomolgus macaques *(Macaca fascicularis),* ZEBOV infection produces a mortality rate of 100% with death occurring 6–8 d following infection with 1,000 pfu [15], while in rhesus macaques *(Macaca mulatta)* ZEBOV produces about 100% mortality with death occurring 7–10 d after infection with 1,000 pfu [16]. In contrast to the guinea pig experiments, the passively transferred polyclonal equine neutralizing IgG described above provided only some minor benefit in the form of a slight delay in the onset of viremia from day 5 to day 7 [13] following ZEBOV challenge of cynomolgus monkeys. No significant reduction in mortality was observed. However, protection against EBOV in primates has been observed in a low dose challenge model. Thus, neutralizing equine IgG protected baboons from <30 LD_50_ (50% lethal dose) ZEBOV challenge when the IgG was given up to 1 h after infection and the serum contained high neutralizing antibody titers (1:128 to 1:512) [17,18], and, similarly, neutralizing ovine serum protected baboons against 0.6 LD_50_ ZEBOV challenge [19].

Here, we studied the ability of passively transferred neutralizing human mAb KZ52 to protect against ZEBOV challenge in rhesus macaques. This passive transfer failed to protect the macaques against challenge with ZEBOV, and, furthermore, had a minimal effect on the explosive viral replication following infection. We showed by ELISA that antibody was present at high levels in serum of the monkeys and that neutralization escape was not responsible for the resistance of virus to antibody prophylaxis.

## Results

### Plasma Viremia in Antibody-Treated Macaques

To evaluate whether IgGl KZ52 could protect against Ebola virus infection in a nonhuman primate animal model, antibody was passively transferred to rhesus macaques followed by challenge with the 1995 ZEBOV (Kikwit) isolate 24 h later. Protection against virus challenge by neutralizing antibodies in naive animals often requires high doses of antibody [2]. Therefore, we used a high dose of 50 mg/kg KZ52, which was close to the maximum practically achievable. In addition, we gave a second bolus of 50 mg/kg of KZ52 on day 4 following infection. The results for the four antibody-treated animals show a steady increase in plasma viremia up to 10^5^–10^7^ pfu/ml on day 7 (Figure 1). These levels of plasma viremia closely parallel those seen in the control animal and typically seen in historical controls [15]. The second bolus of antibody given on day 4 did not appear to have any impact upon the rate of increase of plasma virus (Figure 1). Three of the treated animals were euthanized when moribund at day 9 or 10 post infection. The fourth treated animal showed a decrease in plasma viral load after the peak and survived to day 28 before becoming moribund when it too was euthanized. Although monkey CH46 had less severe symptoms than the other animals in the study, it was concluded that this animal, too, was suffering from disease due to ZEBOV, as evidenced, for example, by copious Ebola virus antigen in the lungs (see below).

**Figure 1:**
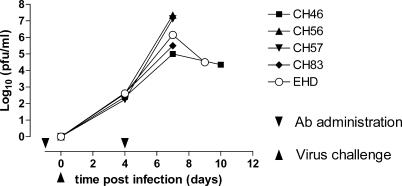
Plasma Viremia in Macaques Challenged with ZEBOV Shown is the measured viremia, in log_10_ pfu per ml, for four antibody-treated monkeys (CH46, CH56, CH57, and CH83) and one untreated control animal (EHD) at days 4, 7, 9, and 10 in plasma by plaque assay as described in Materials and Methods. 50 mg/kg of KZ52 IgG1 human antibody [11] was given intravenously to four rhesus macaques 1 d before and again 4 d after challenge with 1,000 pfu (intramusculary) of the 1995 ZEBOV (Kikwit) isolate. Ab, antibody.

### Serum Antibody Levels in Treated Animals

Serum antibody (KZ52) loads were measured 1 d before virus challenge (day −1) and 4 d after challenge but before antibody boosting (day 4) by an ELISA designed to detect KZ52 as a human antibody that has bound to immobilized ZEBOV glycoprotein. The serum KZ52 antibody levels on day 4 were in the approximate range 200–400 μg/ml (Table 1). The two control monkeys, EHD (untreated and challenged), and a negative monkey (neither treated nor challenged), had very similar background levels of reactivity to the glycoprotein and anti-human antibody as each of the treated monkeys before treatment. A 50-mg/kg dose typically produces serum mAb concentrations in animals on the order of 500 μg/ml after injection [6]. Since the neutralization titer of KZ52 (IC_90_) for ZEBOV is on the order of 0.5–2.5 μg/ml, depending upon the target cell and the presence of complement, the concentrations of KZ52 in the animals at the time of challenge and for the first few days were, as expected, greater than, or on the order of 100 × IC_90_. These concentrations typically provide sterilizing immunity against challenge by a number of viruses [4,20].

**Table 1:**
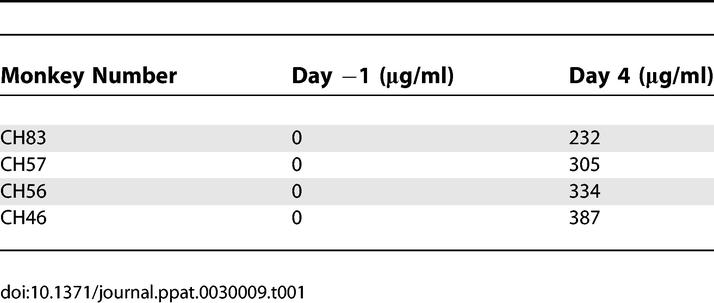
Human KZ52 Antibody Titers in Rhesus Macaques Pre-Treatment 1 d before Challenge with ZEBOV (Day −1) and Pre-Boost 4 d after Challenge with ZEBOV (Day 4)

### Antibody Neutralization of Plasma Virus from Treated Animals

One formal possibility is that the neutralizing antibody has little effect on the course of infection in the treated monkeys because of the rapid emergence of neutralization escape mutants. Accordingly, virus was isolated from a selection of plasma from day 4 (monkeys CH56, CH57, and CH83) and day 7 (monkeys CH56 and CH57). All of these viruses were sensitive to KZ52 so that essentially 100% neutralization was observed in vitro at 40 and 400 μg/ml KZ52 in a plaque assay (see Materials and Methods) using Vero E6 target cells.

### Virus Levels in Different Organs and Immunohistochemistry

In order to gain a better understanding of any differences in pathology between the control and antibody-treated animals, the levels of virus in different organs were surveyed postmortem. Viral levels in the liver, spleen, kidney, adrenal glands, lung, and mesenteric and inguinal lymph nodes were high (10^4^–10^6^ pfu/g) in the control and three of the four treated animals (Table 2). However, monkey CH46, who survived much longer than the other animals (to day 28) showed some major histopathological differences from the other infected monkeys and from the norm for ZEBOV infection [15]. Relatively low viral levels were observed in most of the organs of CH46, and none in the liver, spleen, and adrenal glands. In addition, large immunoreactive monocytes were found in the blood of monkeys CH56, CH57, and CH83, but not in CH46 (Figure 2). Typically, smaller immunoreactive monocytes are seen with ZEBOV infection [15]. The presence of large immunoreactive monocytes may simply reflect uptake of antibody-coated virions via Fc receptors and subsequent viral clearance. However, it is interesting to note that previous studies have implicated mononuclear phagocytes as vehicles for transport of filovirus particles to specific organs such as liver and spleen [21–24]. If virus particles could remain infectious following Fc receptor-mediated uptake in a subset of cells (compare DC-SIGN mediated uptake of HIV-1 by dendritic cells [25]), then the course of disease in monkeys CH56, CH57, and CH83 might represent the net result of inhibition by neutralization and enhancement by antibody-mediated cellular uptake. Interestingly, monkey CH46, who fared somewhat better than the others and lacked virus in the liver and spleen, did not show the presence of large immunoreactive monocytes. This is suggestive of lowered Fc receptor-mediated uptake of virus or reduced activity of the mononuclear phagocytic system.

**Table 2:**
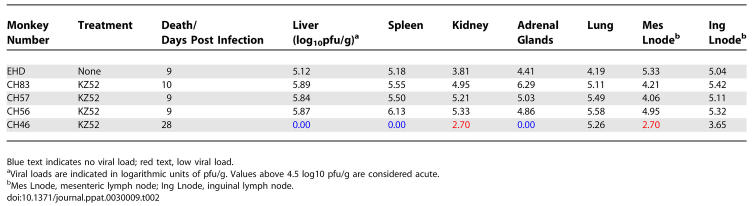
Posthumous Viral Loads in Specific Organs of Rhesus Macaques

**Figure 2:**
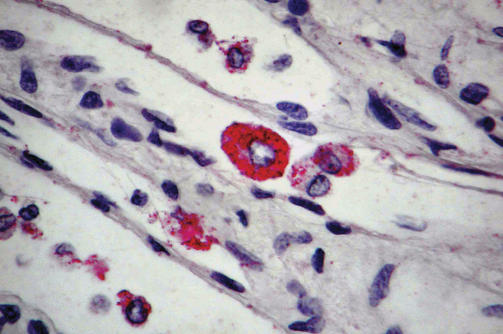
A Large Immunoreactive Monocyte Is Observed in a Blood Vessel in the Kidney of Monkey CH57 Postmortem Similar cells were observed in analysis of monkeys CH83 and CH56. Typically smaller immunoreactive monocytes are seen with ZEBOV infection [15]. Immunohistochemistry was performed as described in Materials and Methods.

## Discussion

Here, we describe the case of a potent neutralizing human monoclonal antibody, administered to give a high serum concentration, which is shown to be unable to protect macaques against challenge with a lethal dose of ZEBOV. The antibody appeared to have very little effect on the course of virus replication or disease in three of four treated animals. Neutralization escape does not appear to explain the lack of protection observed by the antibody. In one animal, a more limited infection was observed, but this macaque did also eventually succumb to the effects of viral disease.

The challenge dose of 1,000 pfu used corresponds to the amount of EBOV contained in a relatively small quantity of fluid (on the order of 1 μl) from an infected individual given the high titers of virus typically found in such individuals (on the order of 10^6^ pfu/ml of blood, for example). Therefore, the challenge dose was not unreasonable in terms of a natural exposure to virus.

The negative results with passive antibody contrast strongly with recent successes in preventing EBOV infection in macaques through vaccination [26,27]. Does this mean that neutralizing antibody is unimportant in vaccine protection? The answer to this question must await further studies. However, a plausible hypothesis to explain all the data would still allow for an important contribution of antibody to vaccine protection. This hypothesis would argue that passive antibody is unable to completely block all EBOV entry to cells, and once a few cells are infected, virus replication is so explosive that it cannot be contained by a de novo generated cellular immune response. Vaccination, on the other hand, will provide CD8+ memory T cells that can be rapidly recruited to become effector cells and limit infection. Certainly, although mAb KZ52 was able to provide protection from disease following ZEBOV challenge in the guinea pig model, immunity was not sterilizing and some viral replication was noted [6]. Since 1 pfu of EBOV is a lethal dose for primates [8], a failure of passive antibody to achieve sterilizing immunity may be critical. Immunohistochemistry, as discussed above, gives some intriguing hints that uptake of antibody-coated virions by monocytes may possibly have a role to play in the course of infection following antibody treatment. We note, however, that previous in vitro studies using isolated human monocytes/macrophages did not find evidence of infectivity-enhancing antibodies [14]. More detailed in vitro and in vivo investigations will be required before any firm conclusions can be drawn. We also note that our experiments were carried out with a single neutralizing monoclonal antibody. It is possible that a more favorable outcome may have been apparent for a combination of neutralizing antibodies or even a combination of neutralizing and nonneutralizing antibodies [2]. However, these possibilities should be weighed against the very high concentrations of neutralizing monoclonal antibody used in the experiments and the efficacy of the antibody in the guinea pig model.

In summary, the inability of high concentrations of neutralizing antibody to even slow viral replication in infected macaques is remarkable and implies a mechanism of infection propagation that is virtually insensitive to antibody. Overall, the results suggest that monoclonal antibody prophylaxis or post-exposure prophylaxis alone are unlikely to be effective strategies in protecting against EBOV, for example, following a needle-stick accident in a research setting or a bioterrorist attack.

## Materials and Methods

### Passive transfer experiment.

50 mg/kg of KZ52 IgG1 human antibody [11] was given intravenously to rhesus macaques (weight, 3.9–4.4 kg) 1 d before challenge (day 0) with 1,000 pfu intramuscularly of the 1995 ZEBOV (Kikwit) isolate and again 4 d later (day +4). One monkey was not given any antibody treatment. The animals were carefully monitored for signs of disease, and Ebola virus plasma viremia was determined at days 4, 7, 9, and 10 in serum by plaque assay as described below.

The investigators adhered to the *Guide for the Care and Use of Laboratory Animals* when conducting research, using animals [28]. The United States Army Medical Research Institute of Infectious Diseases (USAMRIID) animal facilities and animal care and use program are accredited by the Association for Assessment and Accreditation of Laboratory Animal Care International. All infectious material and animals were handled in a maximum-containment biosafety level 4 facility at USAMRIID under standard operating conditions.

### Antibody purification.

IgGl KZ52 was produced and purified as described by Parren et al. [29] and was >98% pure, as determined by sodium dodecyl sulfate-polyacrylamide gel electrophoresis and contained <1 IU of endotoxin/ml, as determined in a quantitative chromagenic *Limulus* amoebecyte lysate assay (BioWhittaker, Cambrex, http://www.cambrex.com).

### Viremia determined by plaque assay.

Plasma viremia and viral loads in organs was determined by virus titration in a conventional plaque assay on Vero E6 cells, as described elsewhere [13,21].

### Neutralization assay.

Samples were diluted into Eagle's minimal essential medium (EMEM; Invitrogen, http://www.invitrogen.com) with 5% heat-inactivated fetal bovine serum (FBS). In the presence and absence of a constant dilution of 1:10 or 1:100 of 4 mg/ml KZ52 (thus, 0.4 mg/ml or 0.04 mg/ml), plasma were titrated from 10^−1^ to 10^−6^ dilutions. Viremia was determined by counting pfu on Vero E6 cell monolayers. Cells grown to confluence in 6-well plates were given 0.2 ml of plasma with and without additional KZ52. The titrated samples were incubated in the presence of KZ52 for 1 h at 37 °C in 5% CO_2_. After absorption, the cells were overlaid with 2 ml of EMEM containing 5% FBS, 25 mM HEPES buffer, 50-μg gentamicin per ml, and 1% agarose. After 10 d, plaques were visible and the cells were removed from the humidified 37 °C incubator to visualize plaques with an inverted phase microscope. 2 ml of neutral red (1:6,000 final concentration; Sigma-Aldrich, http://www.sigmaaldrich.com) was added to each well, and after an additional 24-h incubation, the plaques were counted [11,30].

### ELISA.

Nunc-Immuno Maxisorp enzyme-linked immunosorbent assay (ELISA) plates (Nunc, http://nuncbrand.com) were coated with 100 μl/well of 10 μg/ml lectin from Galanthus Nivalis (Sigma-Aldrich) in PBS and incubated overnight at 4 °C. The plates were then blocked in phosphate-buffered saline (PBS) containing 10% FBS for 2 h. The wells were then washed twice with wash buffer, PBS containing 0.2% Tween 20 (Sigma-Aldrich). 293 cells were transfected with a mammalian expression plasmid coding for transmembrane domain-deleted Ebola glycoprotein. Supernatant (100 μl) from these cells, which contains 0.8–1.3 mg/ml total protein, was used to coat each well for 1 h at room temperature (RT) after the blocking solution was removed from the ELISA plates. Plates were then washed six times with wash buffer. Monkey sera were added in 10-fold dilutions from 1:10 to 1:10^5^ in dilution buffer (PBS containing 1% BSA and 0.02% Tween) and incubated at RT for 1 h. The wells were then washed six times, and a secondary antibody alkaline phosphatase-conjugated goat anti-human immunoglobulin G (IgG) against the F(ab′)_2_ portion of the antibody (Pierce, http://www.piercenet.com) diluted 1:500 was added, and this was incubated for 1 h. Finally, the plates were washed again six times and developed by one tablet of phosphatase substrate (Sigma-Aldrich) in 5 ml of alkaline phosphatase stain buffer (pH 9.8) per plate. The assay was performed as per manufacturer's directions. The plates were read at an optical density of 405 nm on a microplate reader (Molecular Devices, http://www.moleculardevices.com) at 30 min after adding substrate. A panel of normal sera was run each time the assay was performed.

### Immunohistochemistry.

Sections were pretreated with Dako Ready to Use Proteinase K (Dako, http://www.dako.com) for 6 min at RT after deparaffinization and rehydration through a series of graded ethanols. Blocking was performed with Dako's Serum-Free Protein Block for 20 min pre-antibody exposure. The tissue sections were then incubated overnight at 4 °C in primary antibody using an equal mixture of mouse monoclonal antibodies to EBOV GP and VP40 (1:5,000). An alkaline phosphatase-labeled polymer (Dako Envision System, alkaline phosphatase) was incubated on the sections for 30 min, and then color was developed by exposing tissue to 6-bromo-2-hydroxyl-3-naphtholic acid (HistoMark Red; Kikegaard and Perry Laboratories, http://www.kpl.com) substrate for 50 min in the dark. Counterstaining was done with hematoxylin. Positive controls included archived EBOV-infected cynomolgus tissue, and negative controls included replicate sections exposed to anti-Marburg virus antibodies and uninfected cynomolgus macaque tissue [15].

## Abbreviations

EBOV: Ebola virus
IC: inhibitory concentration
LD_50_: 50% lethal dose
mAb: monoclonal antibody
pfu: plaque-forming unit
ZEBOV: *Zaire Ebola* virus
